# Association of Serum Uric Acid With Cardiovascular-Kidney-Metabolic Risk in a Rural Indian Population

**DOI:** 10.1016/j.jacasi.2026.03.006

**Published:** 2026-05-01

**Authors:** Sundaresan Mohanraj, Samrat Ashok Vasudevan, Avinash Kumar Raghupathy, Srinidhi Narayani Seenivasan, Karthika Durairaj, Buvaneswari Gajendran, Parthasarathy Ayothi, Manoj Kumar Baskar, Gowdham Manivel, Madhavi Sambandam, Ganesan Velmurugan, Jeevithan Shanmugam, Mathew Cherian, Thomas Alexander, Krishnan Swaminathan, Arulraj Ramakrishnan

**Affiliations:** a Department of Biochemistry and Microbiology, KMCH Research Foundation, Kovai Medical Center and Hospital, Coimbatore, India; b Department of Biochemistry, Dr NGP Arts and Science College, Coimbatore, India; c Central Research Laboratory, KMCH Institute of Health Sciences and Research, Coimbatore, India; d KMCH College of Nursing, Coimbatore, India; e Department of Community Medicine, KMCH Institute of Health Sciences and Research, Coimbatore, India; f Department of Radiology, Kovai Medical Center and Hospital, Coimbatore, India; g Department of Cardiology, Kovai Medical Center and Hospital, Coimbatore, India; h Liver Unit, Kovai Medical Center and Hospital, Coimbatore, India

**Keywords:** cardiovascular-kidney-metabolic risk, public health, rural population, serum uric acid

Cardiovascular-kidney-metabolic (CKM) syndrome represents a converging epidemic of cardiometabolic and renal disease with major implications for morbidity and mortality.^1^ Contemporary data indicate that most individuals with type 2 diabetes have multiple coexisting CKM conditions, underscoring the systemic and progressive nature of this syndrome.^2^ Chronic kidney disease (CKD) prevalence and mortality continue to rise globally, and cardiovascular disease (CVD) remains the leading cause of death across CKD stages.^3^

The pathways linking metabolic dysfunction, cardiovascular disease, and kidney injury are multifactorial, involving obesity, insulin resistance, dyslipidemia, hypertension, and renal abnormalities.^4^^,^^5^ Altered uric acid metabolism has also been implicated, although evidence for serum uric acid (SUA) as a cardiometabolic marker remains inconsistent.^6^^,^^7^ Our prior work demonstrated divergent associations of SUA with metabolic traits.^8^ Because population-based data from rural South Asia remain limited, we examined the association of SUA with CKM risk in rural Indian adults.

This population-based study used data from the rural Nallampatti Non-communicable Disease (NNCD-II) cohort (Figure 1A).^9^ Adults aged ≥20 years were eligible; pregnant women and individuals younger than 20 years of age were excluded. The study followed the Declaration of Helsinki and was approved by the Institutional Ethics Board of Kovai Medical Center and Hospital (EC/AP/556/08/2017). Written informed consent was obtained.

**Figure 1:**
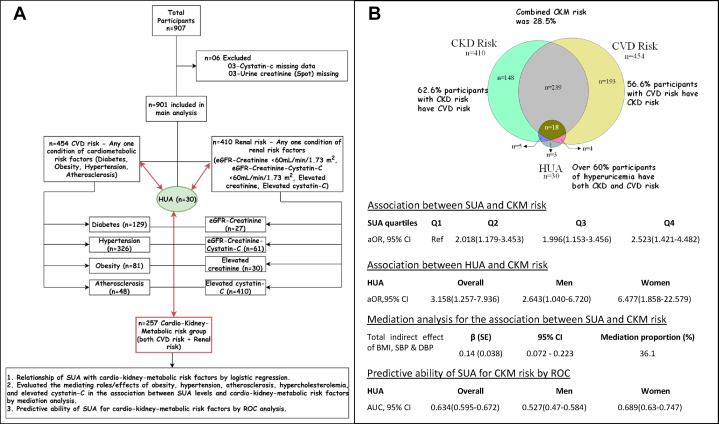
Association Between Serum Uric Acid and Cardio-Kidney-Metabolic Risk (A) Study flowchart. (B) Prevalence of hyperuricemia, cardiovascular disease risk, chronic kidney disease risk, and cardio-kidney-metabolic risk and overview of serum uric acid–cardio-kidney-metabolic association, mediation, and predictive analyses. Logistic regression for binary cardio-kidney-metabolic outcomes; overall adjusted for age, sex, alcohol use, smoking, waist circumference, creatinine clearance, urine creatinine, and total cholesterol; men and women (unadjusted model). aOR = adjusted odds ratio; AUC = area under the curve; BMI = body mass index; CKD = chronic kidney disease; CKM = cardio-kidney-metabolic risk; CVD = cardiovascular disease; DBP = diastolic blood pressure; eGFR = estimated glomerular filtration rate; HUA = hyperuricemia; ROC = receiver-operating characteristic curve; SBP = systolic blood pressure; SUA = serum uric acid.

Standardized questionnaires captured sociodemographic, lifestyle, and medical information. Anthropometry data included weight, height, and waist circumference, and body mass index (BMI) was calculated (kg/m^2^). Blood pressure was measured twice with an automated device and averaged. Blood samples were analyzed for glycosylated hemoglobin (HbA_1c_ by high-performance liquid chromatography), glucose (hexokinase), lipids, SUA, creatinine (automated analyzer), and cystatin C (nephelometry). Carotid intima-media thickness (cIMT) was measured by high-resolution B-mode ultrasonography with radiologist verification.

Overweight status and obesity were defined as BMI ≥ 25 and ≥30 kg/m^2^, respectively. Hypertension was blood pressure ≥ 140/90 mm Hg or antihypertensive use. Diabetes was HbA_1c_ ≥ 6.5% or glucose-lowering therapy. Hyperuricemia was SUA > 6.0 mg/dL (women) or >7.0 mg/dL (men). Kidney measures used sex-specific creatinine and cystatin C thresholds and estimated glomerular filtration rate (eGFR) by the Chronic Kidney Disease Epidemiology Collaboration (CKD-EPI) 2021 equation.^10^ CVD risk was defined as the presence of diabetes, hypertension, obesity, or atherosclerosis (cIMT ≥ 1.0 mm). CKD risk was defined by elevated kidney biomarkers or eGFR < 60 mL/min/1.73 m^2^. CKM risk was defined as the coexistence of both CVD and CKD risk (Figure 1). Prevalence of CVD, CKD, and CKM risk was estimated by cross-tabulation and compared using chi-square test.

SUA and SUA-to-creatinine ratio quartiles were predefined. Continuous variables were summarized as mean ± SD or median (Q1-Q3) and were compared using analysis of variance or Kruskal-Wallis tests with Bonferroni post hoc correction where appropriate; correlations were assessed using Spearman coefficients. Multivariable linear regression was used for continuous CKM traits and logistic regression for binary CKM outcomes, adjusting for age, sex, alcohol use, smoking, waist circumference, creatinine clearance, urine creatinine, and total cholesterol. Sex- and age-stratified analyses were performed. Exploratory mediation analyses were conducted using the PROCESS macro (version 4.2) with 95% CI. Receiver-operating characteristic (ROC) curve analysis evaluated the discriminatory ability of SUA for CKM risk, including sex-stratified analyses. Analyses were performed using IBM-SPSS version 28, and *P* < 0.05 indicated statistical significance.

A total of 901 participants were included: the mean age was 51.9 ± 14.2 years, and 434 of 901 were men (48.2%). Across SUA quartiles, higher SUA was associated with a more adverse cardiometabolic-renal profile. Participants in the highest SUA quartile had a significantly higher prevalence of hypertension, obesity, dyslipidemia, reduced eGFR, elevated creatinine, elevated cystatin-C, and CKM risk, as well as higher waist circumference, BMI, blood pressure, cIMT, and dyslipidemia (all *P* < 0.05). By contrast, higher SUA-to-creatinine ratio quartiles were associated with greater adiposity and dyslipidemia but with lower prevalence of reduced eGFR and elevated creatinine.

The hyperuricemia prevalence was 3.3% (30 of 901; 95% CI: 2.3-4.7), including 4.4% (19 of 434; 95% CI: 2.7-6.8) in men and 2.4% (11 of 467; 95% CI: 1.2-4.2) in women. Overall, 50.4% (454 of 901; 95% CI: 47.1-53.7) had CVD risk, 45.5% (410 of 901; 95% CI: 42.2-48.8) had kidney risk, and 28.5% (257 of 901; 95% CI: 25.6-31.6) had CKM risk. Kidney risk increased across SUA quartiles in both sexes. Among individuals with hyperuricemia, 73.3% (22 of 30; 95% CI: 54.1-87.7) had CVD risk and 76.7% (23 of 30; 95% CI: 57.7-90.1) had kidney risk (both *P* < 0.01). SUA correlated positively with age, adiposity, blood pressure, cIMT, lipids, creatinine, and cystatin-C, and inversely with eGFR and creatinine clearance (*P* < 0.05).

In fully adjusted models (Figure 1), the highest SUA quartile was independently associated with CKM risk (adjusted OR: 2.52; 95% CI: 1.42-4.48; *P* = 0.002). Hyperuricemia was associated with higher odds of CKM risk (adjusted OR: 3.15; 95% CI: 1.25-7.93; *P* < 0.05), with stronger associations in men and older ages. Exploratory mediation analysis showed that BMI and blood pressure partially mediated the SUA-CKM association (indirect effect β = 0.140; 95% CI: 0.072-0.223), jointly accounting for 36.1% of the total effect. In ROC analysis, SUA demonstrated modest discrimination for CKM risk (area under the curve: 0.634; 95% CI: 0.595-0.672; *P* < 0.001), outperforming the SUA/serum creatinine ratio. A SUA cutoff of 4.25 mg/dL yielded 57.2% sensitivity and 60.9% specificity.

In this low-socioeconomic rural Indian population, higher SUA was independently associated with CKM risk. Nearly one-third had combined cardiometabolic and renal risk, with greater burden in the highest SUA quartiles. SUA was associated with hypertension, obesity, dyslipidemia, low eGFR, cystatin-C, and creatinine. Approximately 60% with hyperuricemia had concurrent CKM risk; however, given its low prevalence, these estimates should be interpreted cautiously, and quartile analyses provide a more robust assessment across the SUA distribution.

Exploratory mediation analyses suggested that BMI and blood pressure may partially explain the SUA-CKM association, implicating adiposity and hemodynamic stress as shared pathways. These findings are biologically plausible, given the evidence linking SUA to oxidative stress, endothelial dysfunction, and renin-angiotensin activation, contributing to hypertension and renal injury.^11^ The modest discriminatory performance of SUA indicates limited standalone predictive value, supporting its use within integrated risk assessment in resource-limited rural settings.

This study has limitations. The cross-sectional design precludes causal inference, and reverse causality is possible whereby impaired kidney function may elevate SUA levels. Inclusion of waist circumference and urine creatinine in fully adjusted models may have introduced partial overadjustment. Our CKM construct represents an operational epidemiologic definition rather than the formal American Heart Association/American Diabetes Association staged framework. Additionally, mediation analyses are exploratory, as temporal ordering cannot be verified. Longitudinal studies are warranted to clarify directionality and clinical utility.

Hyperuricemia is an independent risk marker of integrated CKM risk in rural Indian adults and may represent a simple, scalable tool for early cardio-kidney-metabolic risk stratification and prevention.

### Data Availability

Upon reasonable request, the datasets generated during this work are available through correspondence.

## Funding Support and Author Disclosures

The authors have reported that they have no relationships relevant to the contents of this paper to disclose.
